# Crystal structure and Hirshfeld surface analysis of 5-[(5-nitro-1*H*-indazol-1-yl)meth­yl]-3-phenyl-4,5-di­hydro­isoxazole

**DOI:** 10.1107/S2056989018017590

**Published:** 2019-01-01

**Authors:** Mohammed Boulhaoua, Sevgi Kansiz, Mohamed El Hafi, Sanae Lahmidi, Necmi Dege, Mohammed Benchidmi, Joel T. Mague

**Affiliations:** a Laboratoire de Chimie Organique Hétérocyclique, Centre de Recherche Des Sciences des Médicaments, Pôle de Compétence Pharmacochimie, Av Ibn Battouta, BP 1014, Faculté des Sciences, Université Mohammed V, Rabat, Morocco; b Ondokuz Mayıs University, Faculty of Arts and Sciences, Department of Physics, 55139, Kurupelit, Samsun, Turkey; c Department of Chemistry, Tulane University, New Orleans, LA 70118, USA

**Keywords:** crystal structure, indazole, oxazole, hydrogen bond, π–π-stacking, Hirshfeld surface analysis

## Abstract

In the title compound, C_17_H_14_N_4_O_3_, the indazole unit is planar to within 0.0171 (10) Å and makes dihedral angles of 6.50 (6) and 6.79 (4)°, respectively, with the nitro and pendant phenyl groups. The conformation of the oxazole ring is best described as an envelope. In the crystal, oblique stacks along the *a*-axis direction are formed by π–π stacking inter­actions between the indazole unit and the pendant phenyl rings of adjacent mol­ecules. The stacks are linked into pairs through C—H⋯O hydrogen bonds. Hirshfeld surface analysis and two-dimensional fingerprint plots indicate that the most important contributions to the crystal packing are from H⋯H (36.3%), O⋯H/H⋯O (23.4%), C⋯H/H⋯C (13.4%) and N⋯H/H⋯N (11.4%) inter­actions.

## Abstract 2

In the title compound the indazole portion is planar and the nitro group and the pendant phenyl ring are coplanar within 7°. Oblique stacks along the *a*-axis direction are formed by π–π-stacking inter­actions between the indazole unit and the pendant phenyl rings of adjacent mol­ecules. The stacks are linked into pairs through C—H⋯O hydrogen bonds.

## Chemical context   

Indazole derivatives are of pharmaceutical inter­est in a variety of therapeutic areas. They exhibit a variety of biological activities such as HIV protease inhibition (Patel *et al.*, 1999 ▸), anti­arrhythmic and analgesic activities (Mosti *et al.*, 2000 ▸), and anti­tumor activity and anti­hypertensive properties (Bouissane *et al.*, 2006 ▸; Abbassi *et al.*, 2012 ▸). The present work is a continuation of an investigation of indazole derivatives published by our team (Boulhaoua *et al.*, 2015 ▸). In this context, we synthesized the title compound by reaction of benzaldoxime with 1-allyl-5-nitro-1*H*-indazole in a biphasic medium (water–chloro­form). We report herein its crystal and mol­ecular structures along with the Hirshfeld surface analysis.
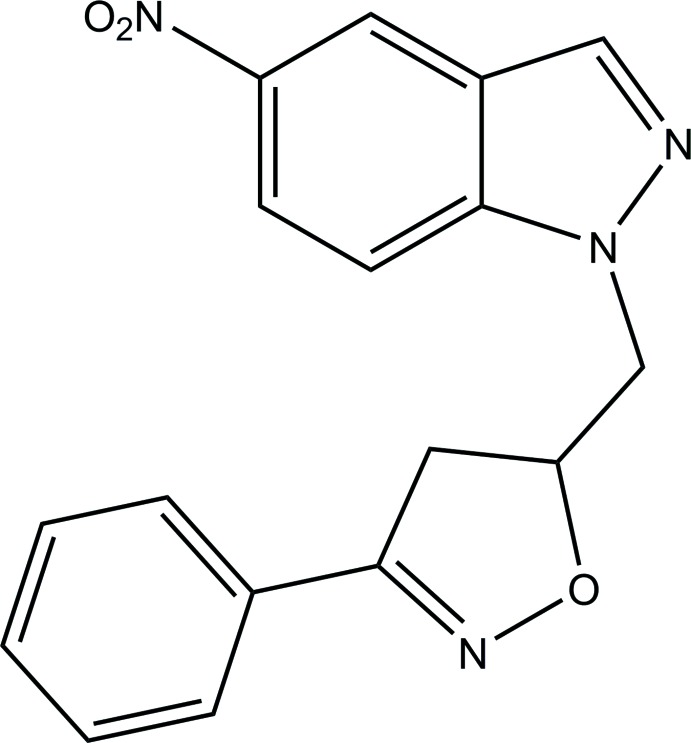


## Structural commentary   

**Figure 1:**
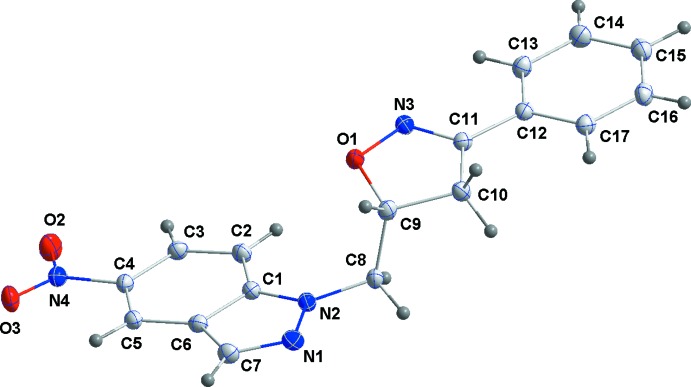
The title mol­ecule with the labelling scheme and 50% probability ellipsoids.

In the title compound (Fig. 1 ▸), the indazole portion is planar to within 0.0171 (10) Å (r.m.s. deviation = 0.0095) with atom C6 the furthest from the mean plane. The nitro group is twisted out of this plane by 6.50 (6)° while the pendant phenyl group makes a dihedral angle of 6.79 (4)° with the plane of the indazole unit. A puckering analysis of the oxazole ring gave parameters *Q*(2) = 0.1499 (12) Å and φ(2) = 325.7 (5)° with the conformation best described as an envelope on C9.

## Supra­molecular features   

**Figure 2:**
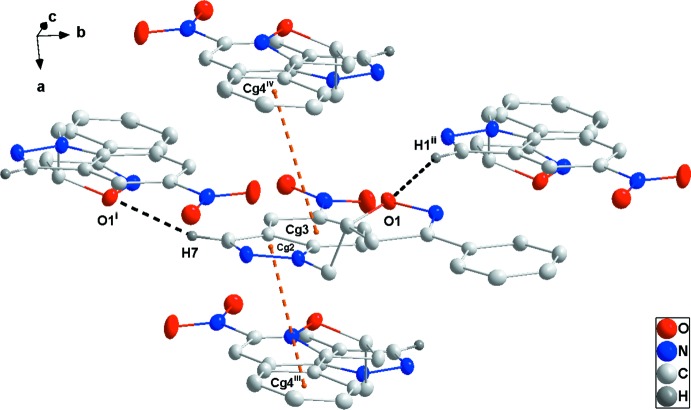
Detail of the inter­molecular C—H⋯O hydrogen bonds (black dashed lines) and π–π-stacking inter­actions (orange dashed lines) [symmetry codes: (i) −*x* + 1, *y* − 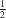, −*z* + 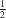; (ii) −*x* + 1, *y* + 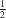, −*z* + 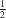; (iii) *x*, −*y* + 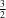, *z* − 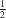; (iv) *x* − 1, −*y* + 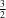, *z* − 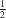; *Cg*2, *Cg*3 and *Cg*4 are the centroids of the C1/C6/C7/N1/N2, C1–C6 and C12–C17 rings, respectively].

In the crystal, the mol­ecules form oblique stacks along the *a*-axis direction through π–π-stacking inter­actions (Fig. 2 ▸) between the five-membered ring of the indazole unit (N1/N2/C1/C6/C7; centroid *Cg*2) and the pendant phenyl ring (C12–C17; centroid *Cg*4) of an adjacent mol­ecule [*Cg*2⋯*Cg*4(*x*, 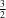 − *y*, −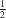 + *z*) = 3.7302 (7) Å; dihedral angle = 3.00 (6)°] and between the six-membered ring of the indazole unit (C1–C6; centroid *Cg*3) and the pendant phenyl ring of a second neighbour [*Cg*3⋯*Cg*4(−1 + *x*, 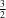 − *y*, −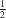 + *z*) = 3.8286 (7) Å; dihedral angle = 3.65 (6)°]. These stacks are associated into pairs through C7—H7⋯O1 hydrogen bonds (Table 1 ▸ and Figs. 2 ▸ and 3 ▸).

## Database survey   

A search of the Cambridge Structural Database (CSD, version 5.39, updates August 2018; Groom *et al.*, 2016 ▸) for the 1-methyl-5-nitro-1*H*-indazole skeleton yielded six hits. In all of these compounds, the indazole rings are planar as in the title compound. In the crystals of all six compounds, mol­ecules are linked by C—H⋯O hydrogen bonds, similar to what is observed in the crystal of the title compound. The N—O bond lengths vary from *ca* 1.213–1.236 Å and the C_aromatic_—NO_2_ bond lengths vary from *ca* 1.456–1.465 Å. In the title compound, the corresponding bond lengths are 1.229 (2), 1.238 (1) and 1.457 (2) Å, respectively. The C_aromatic_-bound nitro group and indazole ring are inclined to each other by a dihedral angle of 4.0 (2)° in AKEFIH (Boulhaoua, El Hafi *et al.*, 2016*b* ▸), 7.0 (9)° in APALOU (Boulhaoua, Essaghouani *et al.*, 2016 ▸), 4.6 (4)° in KEHTEZ (Boulhaoua *et al.*, 2017 ▸), 19.2 (2)° in PUVSOO (Zaleski *et al.*, 1998 ▸), 1.9 (9)° in UJUJOA (Boulhaoua, El Hafi *et al.*, 2016*a* ▸) and 7.9 (5)° in UJUKOB (Boulhaoua, Abdelahi *et al.*, 2016 ▸), compared to 6.5 (6)° in the title compound. Therefore, the various geometrical parameters for the title compound are typical for 1-methyl-5-nitro-1*H*-indazoles.

## Hirshfeld surface analysis   

**Figure 4:**
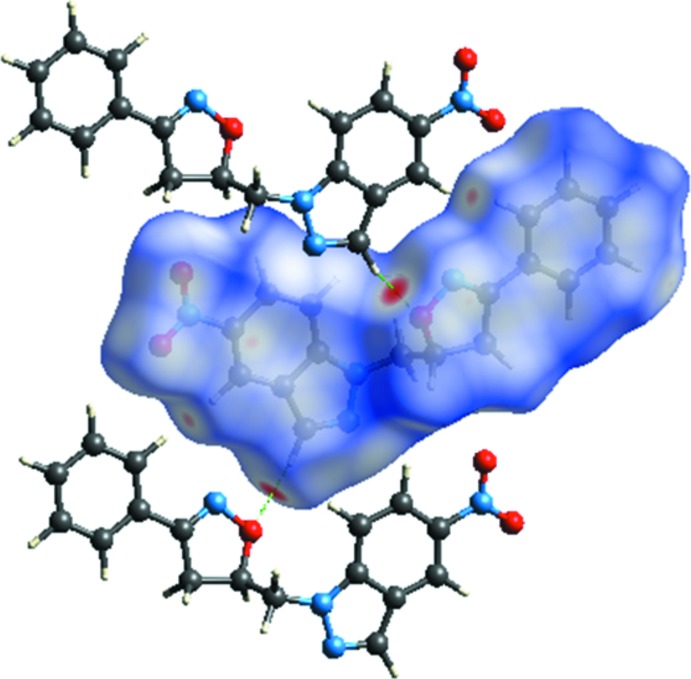
Hirshfeld surface mapped over *d*_norm_ to visualize the inter­molecular inter­actions.

In order to visualize the inter­molecular inter­actions in the crystal of the title compound, a Hirshfeld surface analysis was carried out by using *CrystalExplorer17.5* (Turner *et al.*, 2017 ▸). The *d*_norm_ representation of the Hirshfeld surface reveals the close contacts of the hydrogen-bond donors and acceptors and other close contacts are also evident. The mol­ecular Hirshfeld surfaces were performed using a standard (high) surface resolution with the three-dimensional *d*_norm_ surfaces mapped over a fixed colour scale of −0.191 (red) to 1.051 (blue) Å. The red spots on the surface indicate the inter­molecular contacts involved in the hydrogen bonds. In Fig. 4 ▸, the identified red spot is attributed to the H⋯O close contacts which are due to the C—H⋯O hydrogen bonds (Table 1 ▸).

**Figure 5:**
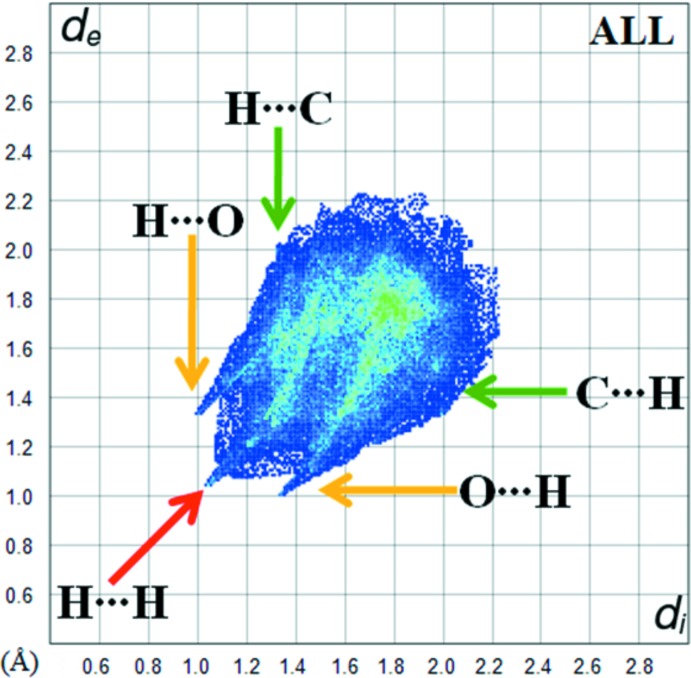
The fingerprint plot for the title compound.

Fig. 5 ▸ shows the two-dimensional fingerprint plot for the sum of the contacts contributing to the Hirshfeld surface represented in normal mode. The O⋯H/H⋯O contacts (23.4%) between the oxygen atoms inside the surface and the hydrogen atoms outside the surface, *d*_e_ + *d*_i_ ∼2.3 Å are shown two symmetrical points at the top, bottom left and right, which are characteristic of C—H⋯O hydrogen bond. The (*d*_i_, *d*_e_) points associated with he H⋯H contacts in this study (36.3%) are characterized by an end point that points to the origin and corresponds to *d*_i_ = *d*_e_ = 1.08 Å. C⋯H/H⋯C and N⋯H/H⋯N inter­actions (13.4% and 11.4%, respectively) are represented by two symmetrical wings on the left and right sides. In addition, the C⋯C (7.5%), C⋯N/N⋯C (4.7%), O⋯C/C⋯O (2.2%) and O⋯N/N⋯O (0.9%) contacts contribute to the Hirshfeld surface.

**Figure 6:**
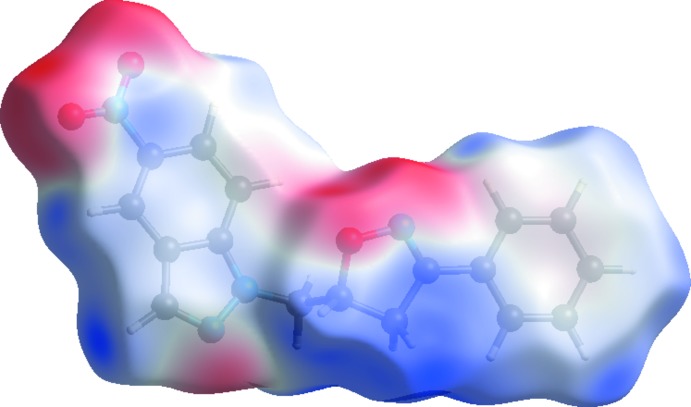
A view of the three-dimensional Hirshfeld surface plotted over mol­ecular electrostatic potential in the range −0.0698 to 0.0535 a.u. using the STO-3 G basis set at the Hartree–Fock level of theory.

A view of the three-dimensional Hirshfeld surface of the title compound plotted over mol­ecular electrostatic potential in the range −0.0698 to 0.0535 a.u. using the STO-3G basis set at the Hartree–Fock level of theory is shown in Fig. 6 ▸. The C—H⋯O hydrogen-bond donors and acceptors are shown as blue and red areas around the atoms related with positive (hydrogen-bond donors) and negative (hydrogen-bond acceptors) electrostatic potentials, respectively.

## Synthesis and crystallization   

To a solution of 1-allyl-5-nitro-1*H*-indazole (0.5 g, 2.46 mmol) and benzaldoxime (4.9 mmol, 0.6 g) in chloro­form (20 mL), a solution of sodium hypochlorite 24% (10 mL) was added dropwise to the mixture and stirred at 273 K for 4h. The resulting mixture was washed with water, dried over MgSO_4_ and the solvent was evaporated under reduced pressure. The residue was then purified by column chromatography on silica gel using a mixture of hexa­ne/ethyl acetate (*v*/*v* = 80/20) as eluent. Colourless crystals were isolated when the solvent was allowed to evaporate (yield: 65%).

## Refinement   

Crystal data, data collection and structure refinement details are summarized in Table 2 ▸. All H atoms were located in a difference-Fourier map and freely refined.

## Supplementary Material

Crystal structure: contains datablock(s) global, I. DOI: 10.1107/S2056989018017590/dx2013sup1.cif

Structure factors: contains datablock(s) I. DOI: 10.1107/S2056989018017590/dx2013Isup2.hkl

Supporting information file. DOI: 10.1107/S2056989018017590/dx2013Isup3.cdx

Supporting information file. DOI: 10.1107/S2056989018017590/dx2013Isup4.cml

CCDC reference: 1884538

Additional supporting information:  crystallographic information; 3D view; checkCIF report

## Figures and Tables

**Figure 3:**
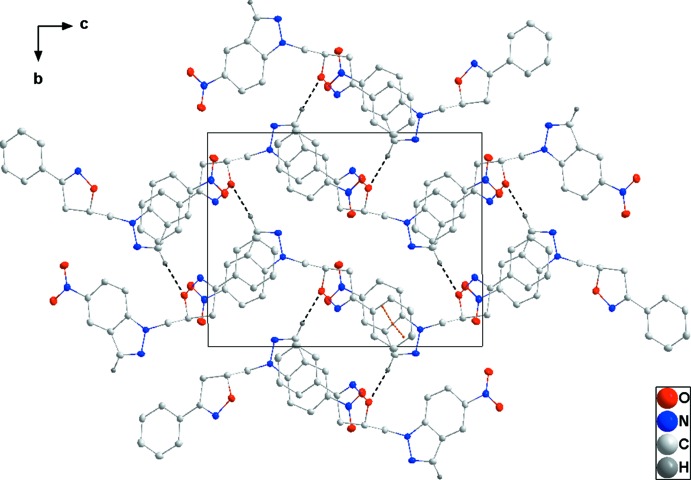
Packing viewed along the *a*-axis direction. A portion of the inter­molecular inter­actions, depicted as in Fig. 2 ▸, is shown.

**Table 1:** Hydrogen-bond geometry (Å, °)

*D*—H⋯*A*	*D*—H	H⋯*A*	*D*⋯*A*	*D*—H⋯*A*
C7—H7⋯O1^i^	0.959 (16)	2.467 (16)	3.3877 (14)	160.9 (13)

Symmetry code: (i) 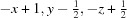.

**Table 2:** Experimental details

Crystal data	
Chemical formula	C_17_H_14_N_4_O_3_
*M*_r_	322.32
Crystal system, space group	Monoclinic, *P*2_1_/*c*
Temperature (K)	100
*a*, *b*, *c* (Å)	7.8595 (4), 11.8831 (7), 15.5716 (9)
β (°)	101.853 (1)
*V* (Å^3^)	1423.30 (14)
*Z*	4
Radiation type	Mo *K*α
μ (mm^−1^)	0.11
Crystal size (mm)	0.35 × 0.32 × 0.17

Data collection	
Diffractometer	Bruker SMART APEX CCD
Absorption correction	Multi-scan (*SADABS*; Bruker, 2016 ▸)
*T*_min_, *T*_max_	0.90, 0.98
No. of measured, independent and observed [*I* > 2σ(*I*)] reflections	26832, 3807, 3116
*R*_int_	0.032
(sin θ/λ)_max_ (Å^−1^)	0.684

Refinement	
*R*[*F* ^2^ > 2σ(*F* ^2^)], *wR*(*F* ^2^), *S*	0.041, 0.120, 1.05
No. of reflections	3807
No. of parameters	273
H-atom treatment	All H-atom parameters refined
Δρ_max_, Δρ_min_ (e Å^−3^)	0.49, −0.20

Computer programs: *APEX3* and *SAINT* (Bruker, 2016 ▸), *SHELXT* (Sheldrick, 2015*a* ▸), *SHELXL2014* (Sheldrick, 2015*b* ▸), *DIAMOND* (Brandenburg & Putz, 2012 ▸) and *SHELXTL* (Sheldrick, 2008 ▸).

## supplementary crystallographic information

### Crystal data

**Table:**

C_17_H_14_N_4_O_3_	*F*(000) = 672
*M**_r_* = 322.32	*D*_x_ = 1.504 Mg m^−^^3^
Monoclinic, *P*2_1_/*c*	Mo *K*α radiation, λ = 0.71073 Å
*a* = 7.8595 (4) Å	Cell parameters from 9953 reflections
*b* = 11.8831 (7) Å	θ = 2.7–29.1°
*c* = 15.5716 (9) Å	µ = 0.11 mm^−^^1^
β = 101.853 (1)°	*T* = 100 K
*V* = 1423.30 (14) Å^3^	Block, colourless
*Z* = 4	0.35 × 0.32 × 0.17 mm

### Data collection

**Table:**

Bruker SMART APEX CCD diffractometer	3807 independent reflections
Radiation source: fine-focus sealed tube	3116 reflections with *I* > 2σ(*I*)
Graphite monochromator	*R*_int_ = 0.032
Detector resolution: 8.3333 pixels mm^-1^	θ_max_ = 29.1°, θ_min_ = 2.2°
φ and ω scans	*h* = −10→10
Absorption correction: multi-scan (*SADABS*; Bruker, 2016)	*k* = −16→16
*T*_min_ = 0.90, *T*_max_ = 0.98	*l* = −21→21
26832 measured reflections	

### Refinement

**Table:**

Refinement on *F*^2^	Primary atom site location: structure-invariant direct methods
Least-squares matrix: full	Secondary atom site location: difference Fourier map
*R*[*F*^2^ > 2σ(*F*^2^)] = 0.041	Hydrogen site location: difference Fourier map
*wR*(*F*^2^) = 0.120	All H-atom parameters refined
*S* = 1.05	*w* = 1/[σ^2^(*F*_o_^2^) + (0.0692*P*)^2^ + 0.3982*P*] where *P* = (*F*_o_^2^ + 2*F*_c_^2^)/3
3807 reflections	(Δ/σ)_max_ < 0.001
273 parameters	Δρ_max_ = 0.49 e Å^−^^3^
0 restraints	Δρ_min_ = −0.20 e Å^−^^3^

### Special details

**Table:**

Experimental. The diffraction data were obtained from 3 sets of 400 frames, each of width 0.5° in ω, colllected at φ = 0.00, 90.00 and 180.00° and 2 sets of 800 frames, each of width 0.45° in φ, collected at ω = –30.00 and 210.00°. The scan time was 20 sec/frame.
Geometry. All esds (except the esd in the dihedral angle between two l.s. planes) are estimated using the full covariance matrix. The cell esds are taken into account individually in the estimation of esds in distances, angles and torsion angles; correlations between esds in cell parameters are only used when they are defined by crystal symmetry. An approximate (isotropic) treatment of cell esds is used for estimating esds involving l.s. planes.
Refinement. Refinement of F^2^ against ALL reflections. The weighted R-factor wR and goodness of fit S are based on F^2^, conventional R-factors R are based on F, with F set to zero for negative F^2^. The threshold expression of F^2^ > 2sigma(F^2^) is used only for calculating R-factors(gt) etc. and is not relevant to the choice of reflections for refinement. R-factors based on F^2^ are statistically about twice as large as those based on F, and R- factors based on ALL data will be even larger.

### Fractional atomic coordinates and isotropic or equivalent isotropic displacement parameters (Å^2^)

**Table:**

	*x*	*y*	*z*	*U*_iso_*/*U*_eq_	
O1	0.65493 (11)	0.74043 (7)	0.41423 (5)	0.0191 (2)	
O2	0.30551 (13)	0.88196 (8)	−0.02290 (6)	0.0285 (2)	
O3	0.20589 (12)	0.71575 (8)	−0.06215 (6)	0.0245 (2)	
N1	0.69220 (13)	0.46778 (8)	0.25680 (7)	0.0185 (2)	
N2	0.72589 (12)	0.57977 (8)	0.27365 (6)	0.0158 (2)	
N3	0.74782 (13)	0.82212 (8)	0.47244 (7)	0.0174 (2)	
N4	0.30425 (13)	0.77874 (9)	−0.01243 (7)	0.0189 (2)	
C1	0.63510 (14)	0.64559 (10)	0.20885 (7)	0.0144 (2)	
C2	0.62565 (15)	0.76290 (10)	0.19937 (8)	0.0169 (2)	
H2	0.685 (2)	0.8130 (14)	0.2417 (11)	0.031 (4)*	
C3	0.51782 (15)	0.80431 (10)	0.12540 (8)	0.0168 (2)	
H3	0.504 (2)	0.8820 (14)	0.1145 (10)	0.024 (4)*	
C4	0.42155 (14)	0.73017 (10)	0.06319 (7)	0.0161 (2)	
C5	0.42849 (14)	0.61464 (10)	0.07119 (8)	0.0158 (2)	
H5	0.357 (2)	0.5659 (14)	0.0287 (10)	0.028 (4)*	
C6	0.53865 (14)	0.57188 (9)	0.14574 (8)	0.0152 (2)	
C7	0.58181 (15)	0.46238 (10)	0.18078 (8)	0.0182 (2)	
H7	0.542 (2)	0.3910 (13)	0.1558 (10)	0.025 (4)*	
C8	0.84131 (15)	0.61131 (10)	0.35495 (7)	0.0171 (2)	
H8A	0.9079 (18)	0.6778 (12)	0.3442 (9)	0.016 (3)*	
H8B	0.9229 (18)	0.5481 (12)	0.3686 (9)	0.016 (3)*	
C9	0.74607 (15)	0.63288 (10)	0.42928 (8)	0.0168 (2)	
H9	0.6526 (19)	0.5757 (13)	0.4289 (10)	0.020 (4)*	
C10	0.87110 (16)	0.64689 (10)	0.51745 (8)	0.0174 (2)	
H10A	0.992 (2)	0.6201 (13)	0.5155 (10)	0.023 (4)*	
H10B	0.829 (2)	0.6065 (13)	0.5660 (11)	0.023 (4)*	
C11	0.86519 (14)	0.77242 (10)	0.52933 (7)	0.0153 (2)	
C12	0.97578 (14)	0.83461 (10)	0.60166 (7)	0.0155 (2)	
C13	0.96755 (15)	0.95199 (10)	0.60656 (8)	0.0184 (2)	
H13	0.890 (2)	0.9941 (14)	0.5607 (11)	0.028 (4)*	
C14	1.06651 (16)	1.00864 (11)	0.67741 (8)	0.0213 (3)	
H14	1.060 (2)	1.0871 (16)	0.6806 (11)	0.035 (5)*	
C15	1.17610 (16)	0.94898 (11)	0.74367 (8)	0.0214 (3)	
H15	1.249 (2)	0.9886 (15)	0.7961 (11)	0.034 (4)*	
C16	1.18699 (16)	0.83273 (11)	0.73863 (8)	0.0199 (2)	
H16	1.256 (2)	0.7904 (14)	0.7837 (10)	0.026 (4)*	
C17	1.08704 (15)	0.77535 (10)	0.66777 (8)	0.0177 (2)	
H17	1.0992 (19)	0.6917 (13)	0.6650 (10)	0.024 (4)*	

### Atomic displacement parameters (Å^2^)

**Table:**

	*U*^11^	*U*^22^	*U*^33^	*U*^12^	*U*^13^	*U*^23^
O1	0.0189 (4)	0.0187 (4)	0.0176 (4)	0.0040 (3)	−0.0014 (3)	−0.0028 (3)
O2	0.0362 (6)	0.0189 (5)	0.0263 (5)	0.0060 (4)	−0.0030 (4)	0.0037 (4)
O3	0.0237 (5)	0.0280 (5)	0.0183 (4)	−0.0012 (4)	−0.0041 (4)	−0.0007 (4)
N1	0.0223 (5)	0.0131 (5)	0.0202 (5)	−0.0003 (4)	0.0045 (4)	−0.0004 (4)
N2	0.0183 (5)	0.0132 (5)	0.0147 (5)	0.0014 (3)	0.0008 (4)	−0.0005 (3)
N3	0.0181 (5)	0.0178 (5)	0.0157 (5)	0.0010 (4)	0.0021 (4)	−0.0012 (4)
N4	0.0194 (5)	0.0205 (5)	0.0159 (5)	0.0032 (4)	0.0016 (4)	0.0003 (4)
C1	0.0145 (5)	0.0151 (5)	0.0135 (5)	0.0005 (4)	0.0026 (4)	−0.0004 (4)
C2	0.0187 (5)	0.0146 (5)	0.0165 (5)	−0.0003 (4)	0.0017 (4)	−0.0022 (4)
C3	0.0181 (5)	0.0141 (5)	0.0181 (6)	0.0015 (4)	0.0035 (4)	0.0002 (4)
C4	0.0146 (5)	0.0196 (5)	0.0135 (5)	0.0024 (4)	0.0018 (4)	0.0010 (4)
C5	0.0144 (5)	0.0178 (5)	0.0150 (5)	−0.0008 (4)	0.0024 (4)	−0.0023 (4)
C6	0.0147 (5)	0.0150 (5)	0.0162 (5)	−0.0010 (4)	0.0044 (4)	−0.0019 (4)
C7	0.0195 (6)	0.0148 (5)	0.0203 (6)	−0.0016 (4)	0.0038 (4)	−0.0013 (4)
C8	0.0162 (5)	0.0184 (5)	0.0148 (5)	0.0019 (4)	−0.0010 (4)	−0.0008 (4)
C9	0.0180 (5)	0.0147 (5)	0.0169 (5)	0.0015 (4)	0.0016 (4)	0.0011 (4)
C10	0.0216 (6)	0.0150 (5)	0.0148 (5)	0.0027 (4)	0.0018 (4)	0.0004 (4)
C11	0.0165 (5)	0.0151 (5)	0.0147 (5)	0.0011 (4)	0.0039 (4)	0.0011 (4)
C12	0.0148 (5)	0.0175 (5)	0.0145 (5)	0.0008 (4)	0.0037 (4)	0.0013 (4)
C13	0.0174 (5)	0.0171 (5)	0.0201 (6)	0.0007 (4)	0.0022 (4)	0.0021 (4)
C14	0.0214 (6)	0.0175 (6)	0.0245 (6)	−0.0020 (4)	0.0034 (5)	−0.0012 (5)
C15	0.0202 (6)	0.0259 (6)	0.0179 (6)	−0.0036 (5)	0.0033 (5)	−0.0020 (5)
C16	0.0188 (6)	0.0256 (6)	0.0146 (5)	0.0005 (5)	0.0016 (4)	0.0033 (4)
C17	0.0193 (6)	0.0178 (5)	0.0160 (6)	0.0023 (4)	0.0039 (4)	0.0025 (4)

### Geometric parameters (Å, º)

**Table:**

O1—N3	1.4230 (13)	C8—C9	1.5238 (16)
O1—C9	1.4603 (14)	C8—H8A	0.981 (15)
O2—N4	1.2377 (14)	C8—H8B	0.982 (15)
O3—N4	1.2294 (13)	C9—C10	1.5245 (17)
N1—C7	1.3176 (16)	C9—H9	1.000 (15)
N1—N2	1.3716 (13)	C10—C11	1.5050 (15)
N2—C1	1.3577 (14)	C10—H10A	1.006 (16)
N2—C8	1.4472 (15)	C10—H10B	1.007 (16)
N3—C11	1.2840 (15)	C11—C12	1.4724 (16)
N4—C4	1.4573 (15)	C12—C17	1.3966 (15)
C1—C2	1.4022 (16)	C12—C13	1.3991 (16)
C1—C6	1.4147 (15)	C13—C14	1.3866 (17)
C2—C3	1.3734 (16)	C13—H13	0.976 (16)
C2—H2	0.940 (17)	C14—C15	1.3943 (18)
C3—C4	1.4093 (16)	C14—H14	0.936 (19)
C3—H3	0.941 (16)	C15—C16	1.3873 (18)
C4—C5	1.3785 (16)	C15—H15	1.011 (18)
C5—C6	1.3936 (16)	C16—C17	1.3941 (17)
C5—H5	0.967 (17)	C16—H16	0.941 (16)
C6—C7	1.4248 (16)	C17—H17	1.000 (16)
C7—H7	0.959 (16)		
			
N3—O1—C9	108.92 (8)	H8A—C8—H8B	107.8 (11)
C7—N1—N2	106.54 (9)	O1—C9—C8	109.07 (9)
C1—N2—N1	111.44 (9)	O1—C9—C10	104.66 (9)
C1—N2—C8	129.81 (10)	C8—C9—C10	112.09 (10)
N1—N2—C8	118.70 (9)	O1—C9—H9	105.0 (9)
C11—N3—O1	109.12 (9)	C8—C9—H9	110.8 (9)
O3—N4—O2	122.78 (10)	C10—C9—H9	114.6 (9)
O3—N4—C4	118.64 (10)	C11—C10—C9	100.86 (9)
O2—N4—C4	118.56 (10)	C11—C10—H10A	111.8 (9)
N2—C1—C2	131.25 (11)	C9—C10—H10A	112.0 (9)
N2—C1—C6	106.53 (10)	C11—C10—H10B	110.9 (9)
C2—C1—C6	122.22 (10)	C9—C10—H10B	111.9 (9)
C3—C2—C1	117.05 (11)	H10A—C10—H10B	109.2 (13)
C3—C2—H2	119.7 (10)	N3—C11—C12	121.64 (10)
C1—C2—H2	123.2 (10)	N3—C11—C10	114.02 (10)
C2—C3—C4	120.28 (11)	C12—C11—C10	124.27 (10)
C2—C3—H3	122.1 (10)	C17—C12—C13	119.43 (11)
C4—C3—H3	117.6 (10)	C17—C12—C11	119.51 (10)
C5—C4—C3	123.69 (11)	C13—C12—C11	121.03 (10)
C5—C4—N4	118.31 (10)	C14—C13—C12	120.18 (11)
C3—C4—N4	117.97 (10)	C14—C13—H13	119.8 (10)
C4—C5—C6	116.40 (10)	C12—C13—H13	120.0 (10)
C4—C5—H5	121.9 (10)	C13—C14—C15	120.15 (12)
C6—C5—H5	121.6 (10)	C13—C14—H14	119.8 (11)
C5—C6—C1	120.36 (10)	C15—C14—H14	120.0 (11)
C5—C6—C7	135.26 (11)	C16—C15—C14	120.02 (12)
C1—C6—C7	104.35 (10)	C16—C15—H15	118.5 (10)
N1—C7—C6	111.14 (10)	C14—C15—H15	121.4 (10)
N1—C7—H7	120.5 (9)	C15—C16—C17	120.03 (11)
C6—C7—H7	128.3 (9)	C15—C16—H16	121.5 (10)
N2—C8—C9	112.99 (10)	C17—C16—H16	118.4 (10)
N2—C8—H8A	108.6 (8)	C16—C17—C12	120.17 (11)
C9—C8—H8A	110.9 (8)	C16—C17—H17	118.5 (9)
N2—C8—H8B	104.8 (8)	C12—C17—H17	121.3 (9)
C9—C8—H8B	111.4 (8)		
			
C7—N1—N2—C1	0.80 (13)	C1—C6—C7—N1	0.01 (13)
C7—N1—N2—C8	178.51 (10)	C1—N2—C8—C9	86.13 (14)
C9—O1—N3—C11	−10.56 (12)	N1—N2—C8—C9	−91.08 (12)
N1—N2—C1—C2	178.29 (11)	N3—O1—C9—C8	−104.57 (10)
C8—N2—C1—C2	0.9 (2)	N3—O1—C9—C10	15.55 (11)
N1—N2—C1—C6	−0.80 (12)	N2—C8—C9—O1	−73.99 (12)
C8—N2—C1—C6	−178.17 (11)	N2—C8—C9—C10	170.59 (9)
N2—C1—C2—C3	−179.03 (11)	O1—C9—C10—C11	−13.98 (11)
C6—C1—C2—C3	−0.06 (16)	C8—C9—C10—C11	104.09 (10)
C1—C2—C3—C4	0.43 (16)	O1—N3—C11—C12	−176.33 (9)
C2—C3—C4—C5	−0.33 (17)	O1—N3—C11—C10	0.66 (13)
C2—C3—C4—N4	177.74 (10)	C9—C10—C11—N3	8.74 (13)
O3—N4—C4—C5	5.90 (15)	C9—C10—C11—C12	−174.36 (10)
O2—N4—C4—C5	−175.49 (10)	N3—C11—C12—C17	172.37 (11)
O3—N4—C4—C3	−172.27 (10)	C10—C11—C12—C17	−4.30 (16)
O2—N4—C4—C3	6.33 (16)	N3—C11—C12—C13	−5.78 (17)
C3—C4—C5—C6	−0.17 (17)	C10—C11—C12—C13	177.55 (11)
N4—C4—C5—C6	−178.23 (10)	C17—C12—C13—C14	−1.29 (17)
C4—C5—C6—C1	0.53 (16)	C11—C12—C13—C14	176.86 (10)
C4—C5—C6—C7	178.17 (12)	C12—C13—C14—C15	0.67 (18)
N2—C1—C6—C5	178.75 (10)	C13—C14—C15—C16	0.31 (18)
C2—C1—C6—C5	−0.44 (17)	C14—C15—C16—C17	−0.66 (18)
N2—C1—C6—C7	0.47 (12)	C15—C16—C17—C12	0.03 (17)
C2—C1—C6—C7	−178.72 (10)	C13—C12—C17—C16	0.95 (17)
N2—N1—C7—C6	−0.48 (13)	C11—C12—C17—C16	−177.23 (10)
C5—C6—C7—N1	−177.89 (12)		

### Hydrogen-bond geometry (Å, º)

**Table:**

*D*—H···*A*	*D*—H	H···*A*	*D*···*A*	*D*—H···*A*
C7—H7···O1^i^	0.959 (16)	2.467 (16)	3.3877 (14)	160.9 (13)

Symmetry code: (i) −*x*+1, *y*−1/2, −*z*+1/2.
